# Objectively measured environmental factors in relation to school travel mode among adolescents: a decision tree analysis

**DOI:** 10.1186/s12966-025-01727-6

**Published:** 2025-03-04

**Authors:** Lena Malnes, Tommy Haugen, Emma Charlott Andersson Nordbø, Andreas Ivarsson, Elin Kolle, Geir Kåre Resaland, Runar Barstad Solberg, Andreas Åvitsland, Sveinung Berntsen

**Affiliations:** 1 Department of Sport Science and Physical Education, Faculty of Health and Sport Sciences, University of Agder, P.O. Box 422, Kristiansand, 4604 Norway; 2 Department of Public Health Science, Norwegian University of Life Sciences, P.O. Box 5003, Ås, NO-1432 Norway; 3 School of Health and Welfare, Halmstad University, Box 823, Halmstad, SE-301 18 Sweden; 4 Department Sports Medicine, Norwegian School of Sport Sciences, P.O. Box 4014, Ullevål Stadion, Oslo, 0806 Norway; 5 Faculty of Teacher Education and Sports, Western Norway University of Applied Sciences, P.O. Box 7030, Bergen, 5020 Norway; 6 Centre for Epidemic Interventions Research, Norwegian Institute for Public Health, P.O. Box 222, Skøyen, Oslo, 0213 Norway; 7 Department of Education and Sport Science, University of Stavanger, P.O. Box 8600, Stavanger, 4036 Norway

**Keywords:** Commuting, Correlates, Cycling, GIS, Predictors, Teenagers, Walking, Youth

## Abstract

**Background:**

Understanding the factors that influence school travel mode choice is essential for promoting active travel among adolescents. Currently, there is a lack of research that effectively investigates the interactions between demographic and environmental factors on travel behavior. We aimed to investigate the associations between various demographic and environmental characteristics and the choice of school travel modes—walking, cycling, or motorized transport—among adolescents, across the winter and summer seasons.

**Methods:**

Data from 1409 Norwegian adolescents, aged 14–15 years, who participated in the School In Motion project were analyzed. Self-reported travel modes and demographic characteristics were collected via questionnaires, while environmental characteristics were determined using Geographic Information Systems (GIS). A decision tree analysis was conducted utilizing the chi-squared automatic interaction detection algorithm to discern patterns in the data. The present study has a cross-sectional design.

**Results:**

During summer, the predominant travel modes were cycling (39%), walking (37%), and motorized transport (24%). Gender was associated with travel mode choices over short distances (< 1.6 km), with girls favoring walking and boys favoring cycling. For longer commutes, steep inclines were associated with reduced walking and cycling, while higher traffic exposure was associated with increased cycling. During winter, walking (50%) was the most common mode, followed by motorized travel (36%) and cycling (14%). Living near peers was associated with increased walking and cycling among girls. For commutes exceeding 2 km, factors such as available bus transit, more streetlights, the absence of steep hills, and higher urban centrality were linked to increased walking and cycling.

**Conclusions:**

The findings indicated a complex pattern of demographic and environmental factors influencing active travel, with environmental factors becoming increasingly important as commuting distances increased. These findings highlight the importance of considering the interactions of various factors to effectively promote active travel, especially for adolescents undertaking longer commutes.

**Trial registration:**

Clinicaltrials.gov ID no: NCT03817047. Registered on: January 25, 2019 (retrospectively registered).

**Supplementary Information:**

The online version contains supplementary material available at 10.1186/s12966-025-01727-6.

## Introduction

Physical activity among children and adolescents is associated with several health benefits [1]. However, most individuals do not meet the international physical activity guidelines [2, 3]. Travel-related physical activity, such as walking and cycling, could be a major contributor to the overall physical activity levels among adolescents [4]. Given that environmental factors can either facilitate or hinder active travel, it is essential to understand their role in shaping travel behavior [5]. With rapid urbanization worldwide [6], knowledge about how the environment can support active travel is particularly important when designing communities that promote physical activity and healthier lifestyles.

Distance is broadly accepted as one of the most consistent and strongest environmental factors affecting active travel [7–10]. However, previous findings regarding how other environmental factors, such as available bus transit, traffic exposure, and population density, affect active travel have been inconsistent [9, 11]. For example, a recent study synthesizing 29 systematic reviews concluded that the association between most characteristics in the environment and physical activity (including travel-related physical activity) was inconsistent between studies [11]. Another umbrella review, focusing on the relationship between the environment and physical activity among children and adolescents [9], reported mixed findings for bus transit availability, street lighting, traffic exposure, and centrality, with studies showing negative, positive, or no associations with active travel.

The relationship between environmental and demographic factors and travel behavior appears to be complex and characterized by several interactions [12–15]. For example, previous findings indicate that distance from home to school [15], geographical context [14], socioeconomic status (SES) [16], gender [17], and transportation modes [18, 19] moderate the effect of environmental factors on active travel. Da Silva and colleagues [16] found that SES affects the association of streetlights and bicycle infrastructure with active travel among adolescents, with street lighting having a more pronounced impact on commuting for those with moderate SES than those with lower or higher SES. Similarly, a study of adolescents in 12 countries showed that the effect of environmental factors, such as bus transit availability, gender, and traffic speed, on travel behavior varied by country of origin, with a positive association in some countries, and inverse or no association in others [14]. Furthermore, while factors affecting walking and cycling are often reported together under active travel, there may be important differences to consider. For instance, cycling appears to have a curvilinear relationship with distance, unlike the linear relationship observed with walking [14, 18, 19]. Studies have also shown that cycling is more sensitive than walking to seasonal variations, especially among children and adolescents [20, 21]. Moreover, Mandic and colleagues [22] found that adolescents report higher safety concerns for cycling than walking. Therefore, investigating different demographic and environmental factors in relation to each other is essential to fully understand their combined impact on active travel, while also considering seasonal differences.

The environmental impact on walking and cycling has been examined among children [9] and adults [23], but less so among adolescents, particularly in the Scandinavian context. Norway’s unique topography, ranging from flat coastal areas to forested, hilly, and mountainous regions [24], may affect travel behavior. In addition, Norway experiences large seasonal variations, from cold, snowy winters with short daylight hours to mild, bright summers [25], which are important factors to consider when investigating active travel. However, previous studies have rarely accounted for seasonal variations [7]. Therefore, this study aimed to explore how demographic and environmental characteristics are associated with walking, cycling, and motorized travel to school among Norwegian adolescents, during the winter and summer seasons.

## Methods

### Study design

The present study has a cross-sectional design that used baseline data from the School In Motion (ScIM) project conducted during the school year 2017–2018. The design and methods for the main project have been reported elsewhere [26, 27]. In summary, 2084 adolescents aged 14–15 years, who were in the 8th grade, consented to participate in the ScIM project. In spring 2017, we collected self-reported data on their travel mode to school using a computerized questionnaire, which was part of the original ScIM test battery. In addition, environmental characteristics were computed using geographic information systems (GIS) analysis of existing spatial data explicitly obtained for the present study. The Norwegian Directorate for Education and Training funded the ScIM project and implementation of an intervention was assessed in an earlier publication [27]. Furthermore, the participants’ parents signed an informed consent form. Parents and pupils were provided with written and oral information about the project and informed that they could withdraw from data collection at any time. The ScIM project received approval from the Norwegian Centre for Research Data in 2016.

### Sample

**Fig. 1:**
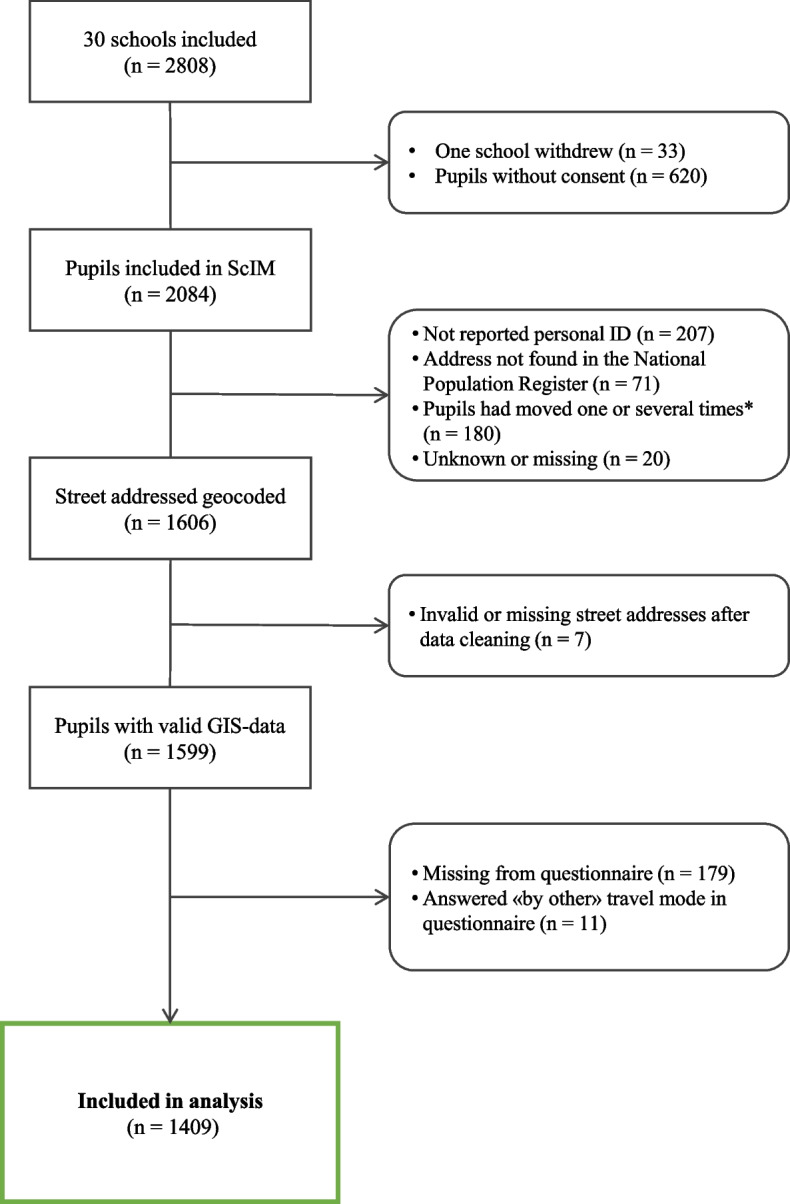
Flow chart of participants included in the analysis. ScIM = the School In Motion study. *N* = number of participants. * = pupils with two or more addresses registered in the National Population Register. Data cleaning involved manually checking the geocoded addresses for validity

The 30 schools involved were located in 18 different municipalities across various parts of Southern Norway, in the regions near each of the four test centers: Norwegian School of Sport Sciences, University of Stavanger, University of Agder and Western Norway University of Applied Sciences. More details about the inclusion and recruitment of the included schools are reported elsewhere [26]. Participants who had provided their full personal identification number and were registered with a valid street address in the National Population Register that could be geocoded using GIS were included. Some participants were registered with multiple addresses and likely moved between the time of data collection in 2017 and data analysis in 2024. Since their residence during the data collection period could not be determined, they were excluded. Consequently, 1409 participants were included in the analysis (Fig. 1).

### Measures

#### Travel mode

Data on school travel mode were collected through a digitalized questionnaire administered during school hours, at the students' respective schools. A researcher was present during data collection to provide assistance and address any questions. The questionnaire items on travel mode were adapted from the Health Behaviour in School-aged Children (HBSC) study [28]. Both trip direction and season may affect physical activity and active travel [29], and given the significant seasonal weather variations in Nordic countries, we incorporated monthly and seasonal specifications into the questionnaire items. Therefore, the participants answered the following question(s); “On a typical day in the spring/summer (April to September) is the main part of your journey to school made by …?” followed by a question capturing travel mode to school during the autumn/winter season. The participants answered with the following options: by walking, by bicycle, by car/motorcycle/moped, by bus/trains/subway/ferry, or by other. Due to the small sample size of participants travelling by car and bus during spring/summer season (*n* = 57 and 270, respectively) and during autumn/winter season (*n* = 168 and 343, respectively), we merged participants traveling by car and bus into one category, motorized travel. In a previous study we investigated the convergent validity of the HBSC items within a subsample of the ScIM project [30]. In summary, the agreement between the HBSC items and a 5-day travel diary during a typical week in the spring was 78%, with a Cohen’s κ of 0.63.

#### Street addresses and geocoding

Personal identification numbers of the pupils were obtained through a consent form. In collaboration with Statistics Norway, we collected the home street addresses using the personal IDs obtained from the National Population Register, and these addresses were geocoded using QGIS, version 3.32.3-Lima (QGIS, 2024) into geographic coordinates. The geographic coordinates were then manually checked for validity using OpenStreetMap (OpenStreetMap contributors, 2023) and cleaned for duplicates and invalid street addresses.

#### Operationalization of factors affecting school travel modes

The three individual variables included in the analysis were gender collected through a questionnaire, and fathers’ and mothers’ educational levels retrieved from Statistics Norway. Some data was collected along the shortest route to school, although others were measured within a line-based neighborhood buffer (as specified below). The line-based neighborhood buffer was operationalized as a 500 m × 50 m line-based road network buffer from participants' residential addresses, based on Oliver et al. [31]. This buffer follows the road network, extending 50 m on both sides (total width 100 m). The road network dataset, sourced from the Norwegian Mapping Authority, included drivable roads, field roads, hiking trails, bicycle paths, and pedestrian walkways, to ensure all potential travel routes for participants were included. The line-based road network buffer was chosen over circular or polygon buffers as it better represents the environment participants can realistically travel withing, aligning with findings by Oliver and colleagues, who reported significant differences in factors associated with walking when comparing different buffer methods [31]. All spatial data were computed using QGIS.

Route based measures:

- *Distance to school* was operationalized as the shortest possible route through the road network, which have shown to be more precise than Euclidean distance [32]. We used data on the road network, including bicycle paths, pedestrian walkways, field roads, and hiking trails, obtained from the Norwegian Mapping Authority. A tolerance of 2 m was set.
- *Steepest hill* was calculated as the steepest incline in percent, within a 10-m radius along the shortest route to or from school. Road gradient data was downloaded from the National Road Database of Norway (Nasjonal vegdatabank) through the website Vegkart.no, and the highest absolute value, regardless of direction, was selected.

Buffer based measures:

- *Pedestrian infrastructure*: Sum of pedestrian path lengths divided by the length of drivable roads within the line-based neighborhood buffer. Pedestrian paths included sidewalks, footpaths, pedestrian and cycling lanes, bicycle lanes, stairs, and pedestrian streets and was obtained from the National Mapping Authority. This operationalization has been employed by other studies [33].
- *Streetlights*: Number of streetlights per 100-m road lengths within the line-based neighborhood buffer, aligning with methods used in previous studies [33].
- *Bus transit availability*: Count of transit stops within the line-based neighborhood buffer, based on data obtained from the Norwegian Public Roads Administration, as used by other studies [33].
- *Traffic exposure*: Average automobile traffic per day passing through the line-based neighborhood buffer, calculated using annual average daily traffic (AADT) from the Norwegian Public Roads Administration. If several estimates of AADT existed within a participant’s neighborhood buffer, the maximum value AADT was used. This score has been used by other studies [34].
- *Living close to peers*: Number of other adolescent participants living within the 500-m line-based neighborhood buffer from the participant’s residence.

Other:

- *Population density*. Based on the 250 m × 250 m population grid from year 2017 from Statistics Norway. The total population in the grid in which each participant resided represents the population density.
- *Centrality index*: Retrieved at the municipal level, based on Statistics Norway’s classification of centrality [35]. As described in earlier publications [36], Statistics Norway [35] classifies municipalities based on their degree of centrality, where a higher index indicates a higher degree of centrality, using the capital of Norway as the benchmark for the most urban score (1000).

### Statistical analysis

Descriptive characteristics are presented as median (interquartile range, IQR) or % (n). We performed a decision tree analysis using the chi-squared automatic interaction detection (CHAID) algorithm to investigate the relationship between independent and dependent variables, stratified by season. All independent variables were included in the model.

Compared to other algorithms, such as CRT or QUEST, CHAID has the advantage of splitting into non-binary nodes making it particular suitable for investigating non-linear relationships and interactions [37]. For model validation, we ran the analyses with different algorithms. The results showed that the CHAID algorithm achieved a higher overall classification accuracy of 71% compared to the CRT (67%) and the QUEST algorithm (55%) using the same criteria. For seasonal accuracy, the CHAID model achieved 71% and 73% percent correct predictions in both seasons, and a tenfold cross-validation risk estimates of 0.298 (SE = 0.012) and 0.285 (SE = 0.012), for summer and winter model respectively. Full classification tables, syntax and SPSS output are available in the supplementary material (Appendix A).

The CHAID algorithm identifies significant predictors of walking, cycling, or motorized travel by splitting the data into homogeneous groups using Chi-square and Bonferroni corrections of significance level to adjust for multiple testing [38]. Continuous variables are split into intervals and iteratively merged if not meeting the significance criteria (set at 0.05 in our study). Because CHAID uses a significance-based splitting criterion from the outset, rather than growing large trees and then pruning them, it is generally less prone to overfitting compared to methods such as CRT and QUEST. To further minimize the risk of overfitting, the maximum tree depth was set at three layers, with a parent node tolerance of 100 and a child node tolerance of 50. Given that the CHAID algorithm includes missing values when constructing the tree, we validated our findings by performing an analysis on a sample consisting of participants without missing values. The main results from this analysis were in line with the results from the analysis with all participants. Furthermore, because the inclusion of GIS data resulted in a smaller sample compared to the main ScIM sample, we conducted a sensitivity analysis, which showed no significant differences between the included and main sample in ScIM (Appendix B). Multicollinearity is not a concern for the CHAID method, but to understand the relationship between the independent variables, we have provided a supplementary table with correlation coefficients using Spearman’s rho (Appendix C). Seasons were defined based on the questionnaire, as spring/summer (April–September) and autumn/winter (October–February).

The data were analyzed using Statistical Package for Social Sciences software (IBM SPSS Statistics for Windows, Version 22.0. Armonk, NY: IBM Corp. USA), and the significance level was set to *p* < 0.05.

## Results

During the summer season, the most common modes of travel were walking (39%) and cycling (37%), whereas, in the winter season, walking (50%) and motorized travel (36%) were most common (Table 1). Participants using motorized transportation generally lived, by median, further from school; resided in areas that had steeper hills, fewer streetlights, and were more rural; and had less pedestrian infrastructure in their neighborhoods than participants who walked or cycled (Table 1).

**Table 1:** Characteristics of the sample categorized by travel mode to school divided by season. Data are presented as median (IQR) for environmental characteristics and percent (n) for individual characteristics

	Summer season			Winter season		
	Walk (*n* = 554)	Cycle (*n* = 528)	Motorized travel (*n* = 337)	Walk (*n* = 707)	Cycle (*n* = 191)	Motorized travel (*n* = 511)
Individual characteristics						
Gender, girls % (n)	62 (342)	37 (193)	55 (179)	53 (371)	34 (64)	56 (283)
University education, % (n)						
Father	52 (269)	57 (284)	46 (145)	50 (353)	58 (111)	45 (231)
Mother	59 (318)	63 (624)	55 (176)	59 (406)	68 (124)	57 (285)
Environmental characteristics						
Distance, km	1.04 (0.78)	1.72 (1.15)	4.91 (4.01)	1.09 (0.86)	1.53 (1.15)	3.33 (3.47)
Steepest hill, % incline	6.9 (4.3)	7.7 (4.8)	10.7 (12.5)	6.9 (4.2)	8.1 (4.5)	10.1 (9.9)
Population, count	192 (182)	137 (166)	89 (156)	183 (176)	204 (157)	89 (139)
Centrality index (0–1000)	890 (47)	890 (66)	848 (99)	890 (66)	890 (0)	848 (118)
Bus transit availability, count	3 (6)	3 (4)	3 (4)	2.5 (6)	5 (6)	2 (4)
Streetlights, count per 100 m	35 (74)	32 (55)	20 (44)	34 (69)	36 (65)	24 (48)
Living close to peers, count	9 (9)	6 (8)	3 (5)	8 (9)	8 (9)	3 (6)
Pedestrian, % road length	37 (31)	35 (30)	24 (29)	35 (28)	42 (32)	26 (30)
Traffic exposure, AADT	6000 (5643)	7000 (9317)	4000 (5400)	6461 (6900)	8200 (9436)	4500 (5900)

Bus stops, streetlights, living close to peers, pedestrian infrastructure, and traffic exposure were measured within a line-based neighborhood buffer of 500 m × 50 m radius from the home address

*km* kilometers, *m* meters, *AADT* annual average daily traffic

**Fig. 2:**
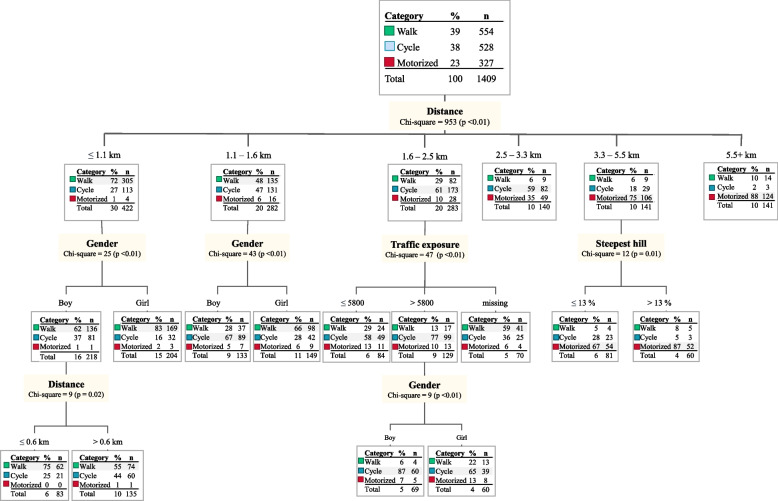
Factors influencing summer season travel mode to school. The CHAID decision tree illustrates environmental and individual factors associated with walking, cycling, or motorized transport during the summer season. The dominant mode is highlighted with shaded text. Traffic exposure is shown as average vehicles per day. * Includes missing values

**Fig. 3:**
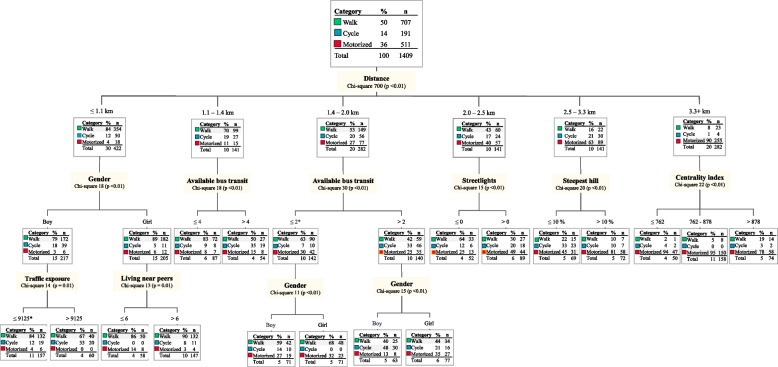
Factors influencing winter travel mode to school. The CHAID decision tree illustrates environmental and individual factors associated with walking, cycling, or motorized transport during the winter season. The dominant mode is highlighted with shaded text. Traffic exposure is shown as vehicles passing per day. * Includes missing values

Distance was the first split in the decision tree analysis and was strongest associated with school travel mode (Figs. 2 and 3). During spring–summer season (Fig. 2), walking was the dominant mode for participants living closest to school. Cycling was most common for those living between 1.6 and 3.3 km from school, while motorized travel was most prevalent among those with the longest commutes. Gender differences were evident for those living closest to school, with girls more likely to walk and less likely to cycle compared to boys. For participants living further away, steep hills were associated with decreased walking and cycling, whereas traffic exposure was positively associated with cycling.

In the autumn–winter season (Fig. 3), walking was dominant for those living within 2.5 km of the school, while motorized travel was more common for those commuting from greater distances. Among participants living 1–2 km from school, bus transit availability was associated with a higher proportion of motorized travel and cycling, although this varied by gender. More streetlights, the absence of a steep hill, and higher urban centrality were associated with increased walking and cycling for participants with longer commuting distances (> 2.0 km). Among participants living closer to school, gender was a factor, where girls walked more and cycled less than boys. Traffic exposure was associated with increased cycling and decreased walking for boys living close to school as illustrated in Fig. 3. Additionally, living near peers was associated with increased walking and cycling among girls with shorter commutes.

## Discussion

This study aimed to investigate the relationship of different environmental and demographic factors with walking, cycling, and motorized travel to school during the summer and winter seasons. The findings indicated a complex pattern of significant associations between travel mode, distance, gender, and environmental features. Distance emerged as the strongest factor associated with travel mode across both seasons, which is supported by findings in previous research [7, 9]. Furthermore, while gender and living close to peers were identified as significant factors for participants living close to school, environmental features, such as available bus, hills, traffic exposure, streetlights, and centrality index, were significant factors for participants living further away from school, although their impact varied by season.

Similar to the findings of Mandic et al. [19], we found that the environment is more important with increased distance from school. The authors investigated self-reported barriers to active travel among 341 adolescents in New Zealand and observed that barriers among parents related to hills and safety increased with increasing commuting distance. Notably, in the study by Mandic et al. [19] environmental variables were self-reported in their study, while we included objectively measured environmental features calculated using GIS in the present study. In addition, their study was conducted in New Zealand [19], and as highlighted by others [14, 39], environmental features that influence travel mode may differ across countries.

Several studies have investigated the ideal or feasible distance for which adolescents can walk and cycle to school and these studies showed large heterogeneity in the results [7, 40]. The heterogeneity between studies may partly be explained by differences in the context in which the studies were conducted, because the feasible distance may vary based on steep hills along the route and season of the year, as illustrated by our findings. Galan et al. [7] mentioned in their review that the feasible distance for active travel could be country- or culture-specific. Other studies have reported that the average distance traveled actively by children increases with age [40]; which also is important to consider when comparing findings between studies. Among the adolescents in the present study, walking was the dominant mode of travel for a distance to school of < 1.6 km and < 2.5 km in the summer and winter seasons, respectively. In the New Zealand study of Mandic et al. [19], the authors categorized participants based on distance, and defined ≤ 2.3 km as walkable, 2.3–4.0 km as cyclable, and > 4.0 km as beyond cyclable.

Seasonal variations in active travel behavior were observed in the present study, with cycling significantly declining in winter. This finding is supported by a previous study that found cycling to be more sensitive to weather variations than walking [20]. Moreover, steep hills along the route were found to be associated with decreased cycling and more walking among participants living > 2.5 km away from school. According to Timperio et al., [41] a steep hill incline of 10% was associated with a reduced likelihood of active travel among children attending elementary school in Australia. Furthermore, a previous study indicated that distance is perceived to be longer if a steep hill is present along the route [42], which may reflect the higher effort required when walking and cycling uphill. Other studies among adults have illustrated that cyclists are more willing to travel longer distances to avoid climbing steep hills along the route [43]. This may be related to our findings where the steepest hill was only a factor for adolescents living < 2.5 km from school. Moreover, we also observed a positive association between cycling and traffic exposure for adolescents living further away from school. Given that cycling has been associated with more safety concerns among parents [19] and adolescents [15], this finding was surprising. One plausible explanation is that cycling requires a higher level of traffic skills and that adolescents who usually cycle longer distances to school are more skilled at navigating in traffic, or that there may be underlying factors moderating this association not accounted for in the present study.

While the findings in the present paper align with previous research, certain unexpected results warrant further exploration. First, gender and living close to peers were only associated with travel mode for the participants with shorter travel distances to school. This finding could be related to the notion that living close to school reduces the logistical barriers to travel [15] and therefore makes gender and living close to peers more prominent factors. Regarding the seasonal variations, we found that the presence of streetlights, centrality index, available bus transit and living close to peers were identified as influential factors only during the winter season. Winter weather conditions, such as snow, icy roads, cold temperatures, and limited daylight, may be an explanation for the observed seasonal differences. In rural areas, roads may be less accessible, due to less efficient snow removal or road maintenance, which have been highlighted as important factors for cycling and walking in qualitative interviews with adults [44].

While it is recommended to promote active travel at different levels of influence, initiatives undertaken at the social or individual level may be less effective if the environment does not support travel behavior among the target population. Consequently, the environment may serve as both a facilitator of and barrier to active travel. Therefore, the present study has implications for policymakers, city planners, and researchers interested in promoting active travel. Moreover, given that rapid urbanization of the population worldwide is increasing the demand for city planning, these findings can contribute to better facilitation and planning for healthy, active living environments for future generations.

### Study strengths and limitations

One study strength is the large sample size from different parts of Southern Norway, which enhances the representativeness of Norwegian adolescents. However, due to the small sample size for car travel, we merged participants traveling by car and bus into one category, motorized travel. This is a limitation due to the potential differences in predictors for public transport and private vehicles, which are not assessed in the present study. Furthermore, we used self-reported travel mode where the participants reported their usual mode of travel to school, which is prone to recall bias. However, one study strength is the use of a widely adopted questionnaire item, which strengthens comparability between studies. The usual mode of travel is the most dominant operationalization of active travel [45, 46], although specifying seasons appear to be less common. Another strength is that we accounted for seasonality and found several seasonal variations, highlighting the importance of considering these differences. Additionally, we validated our questionnaire on a subsample of a previous study [30], which according to a systematic review [45] has not been common in research on active travel among children and adolescents. Another strength of the present study is the objectively measured environmental characteristics using GIS and the retrieval of register data on parental education, which prevents bias related to self-reported data, such as recall bias or social desirability bias. Moreover, the present study has a cross-sectional design, which limits the possibility of investigating causal relationships. Future studies with a longitudinal study design are needed to further investigate the relationship between environmental factors and travel behavior among adolescents.

A strength of this study is the use of decision tree analysis, which is rarely applied in this field and offers a novel perspective. The CHAID algorithm has the ability to perform non-binary splits and efficiently explore interactions that set it apart from other methods such as CART or QUEST [47]. However, the predictive accuracy of our models was 71% and 73% for the summer and winter model, respectively, suggesting that other factors influencing factors of adolescents’ travel mode should be considered. Psychosocial factors, the social environment and social interactions have been associated with active travel among children [7, 13]. Additionally, the models seemed less able to predict cycling compared to walking and motorised travel (Appendix A). Considering Mandic et al. [22], who found that cycling received less social support compared to walking, including measures related to social factors could potentially have improved the model performance; however, such data were unavailable in the present study. Future research should consider integrating these aspects to provide a more comprehensive understanding of the factors influencing active travel. Furthermore, while ensemble tree methods, such as random forest, may offer higher predictive accuracy and additional insights, they are also more complex and less interpretable. Future studies could explore these approaches to complement our findings and further advance the field. Also, a limitation of our analysis is that the CHAID algorithm does not account for clustering in nested samples, unlike multilevel logistic analysis.

## Conclusions

The findings of the present study illustrate a complex relationship between travel mode choices and different environmental and demographical factors among Norwegian adolescents. While distance had the strongest association with travel mode, we observed considerable differences in factors between adolescents living closer to school and those living further away, where environmental barriers, like steep hills and traffic exposure increased with increasing distance. Understanding these nuanced relationships could be crucial for effective policymaking and the design of supportive environments that encourage walking and cycling to school.

## Supplementary Information

Supplementary Material 1

Supplementary Material 2

Supplementary Material 3

## Data Availability

No datasets were generated or analysed during the current study.

## Abbreviations

AADT: Annual Average Daily Traffic
CHAID: Chi-squared Automatic Interaction Detection
GIS: Geographic Information Systems
HBSC: Health Behaviour in School-aged Children
IQR: Interquartile Range
ScIM: School In Motion
SES: Socioeconomic Status
SPSS: Statistical Package for Social Sciences
