# Supplementary material for: Objectively measured environmental factors in relation to school travel mode among adolescents: a decision tree analysis
Source: Int J Behav Nutr Phys Act. 2025 Mar 4;22:26. doi: 10.1186/s12966-025-01727-6 (PMC11877687; doi:10.1186/s12966-025-01727-6)
Supplement: Supplementary file 1 — Supplementary Material 1 [file 12966_2025_1727_MOESM1_ESM.docx]

**Appendix A: Syntax and SPSS output for Decision tree models (CHAID) during spring/summer (Q7) and autumn/winter (Q9).**

1. **Syntax for Decision Tree analysis using CHAID algorithm**

| **Syntax for summer model** | **Syntax for winter model** |
| --- | --- |
| TREE Q7_outcome [n] BY Gender [n] Education_father [n] Education_mother [n] Distance_Road [s]  Steepest_hill [s] Pedestrian_share [s] Streetlights [s] Count_busstop_buffer [s]  Trafic_exposure_max [s] Peers_nearby [s] Rural_Urban_index [s] Population [s]  /TREE DISPLAY=TOPDOWN NODES=STATISTICS BRANCHSTATISTICS=YES NODEDEFS=YES SCALE=AUTO  /DEPCATEGORIES USEVALUES=[1.00 2.00 3.00]  /PRINT MODELSUMMARY CLASSIFICATION RISK  /METHOD TYPE=CHAID  /GROWTHLIMIT MAXDEPTH=3 MINPARENTSIZE=100 MINCHILDSIZE=50  /VALIDATION TYPE=CROSSVALIDATION(10) OUTPUT=BOTHSAMPLES  /CHAID ALPHASPLIT=0.05 ALPHAMERGE=0.05 SPLITMERGED=NO CHISQUARE=PEARSON CONVERGE=0.001  MAXITERATIONS=100 ADJUST=BONFERRONI INTERVALS=10  /COSTS EQUAL  /MISSING NOMINALMISSING=MISSING. | TREE Q9_outcome [n] BY Gender [n] Education_father [n] Education_mother [n] Distance_Road [s]  Steepest_hill [s] Pedestrian_share [s] Streetlights [s] Count_busstop_buffer [s]  Trafic_exposure_max [s] Peers_nearby [s] Rural_Urban_index [s] Population [s]  /TREE DISPLAY=TOPDOWN NODES=STATISTICS BRANCHSTATISTICS=YES NODEDEFS=YES SCALE=AUTO  /DEPCATEGORIES USEVALUES=[1.00 2.00 3.00]  /PRINT MODELSUMMARY CLASSIFICATION RISK  /METHOD TYPE=CHAID  /GROWTHLIMIT MAXDEPTH=3 MINPARENTSIZE=100 MINCHILDSIZE=50  /VALIDATION TYPE=CROSSVALIDATION(10) OUTPUT=BOTHSAMPLES  /CHAID ALPHASPLIT=0.05 ALPHAMERGE=0.05 SPLITMERGED=NO CHISQUARE=PEARSON CONVERGE=0.001  MAXITERATIONS=100 ADJUST=BONFERRONI INTERVALS=10  /COSTS EQUAL  /MISSING NOMINALMISSING=MISSING. |

**2.0 Variable explanation (1 dependent and 12 independent variables per model)**

| **Variable** | **Variable name in SPSS** |
| --- | --- |
| Travel mode summer season | Q7_outcome |
| Travel mode winter season | Q9_outcome |
| Gender | Gender |
| Parental education father | Education_father |
| Parental education mother | Education_mother |
| Distance to school | Distance_Road |
| Steep hill along route | Steepest_hill |
| Pedestrian infrastructure | Pedestrian_share |
| Streetlights in neighbourhood | Streetlights |
| Bus transit availability | Count_busstop_buffer |
| Traffic Exposure | Trafic_exposure_max |
| Living close to peers | Peers_nearby |
| Centrality Index | Rural_Urban_index |
| Population density | Population |

**3.0 SPSS output**

**3.1 Results for model - summer season (Q7_outcome)**


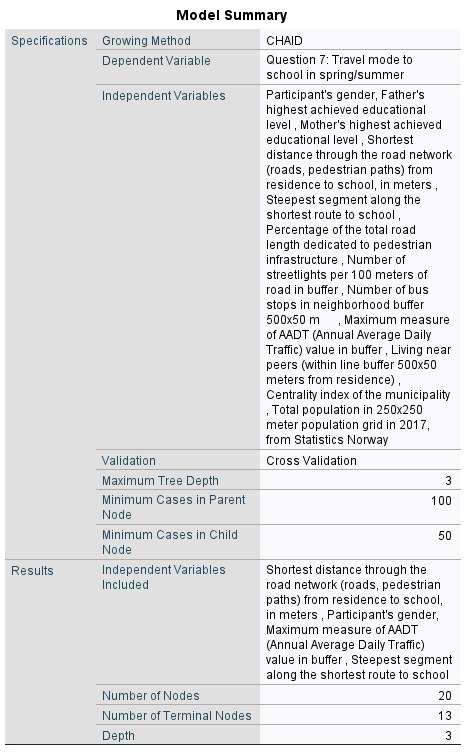

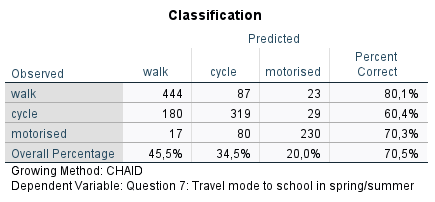

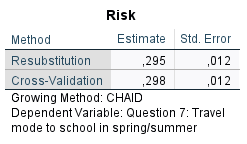


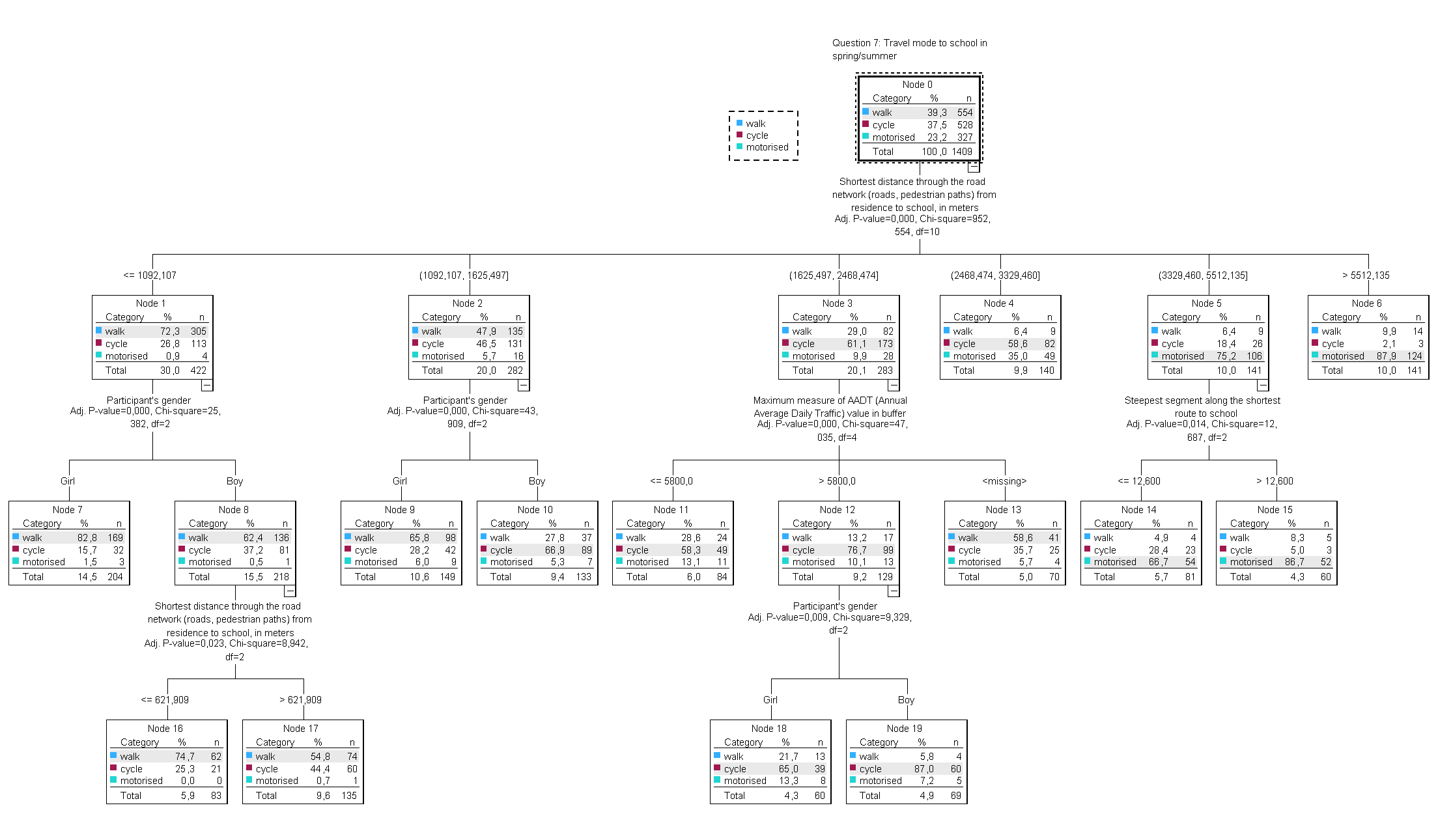


**3.2 Results for model - winter season (Q9_outcome)**


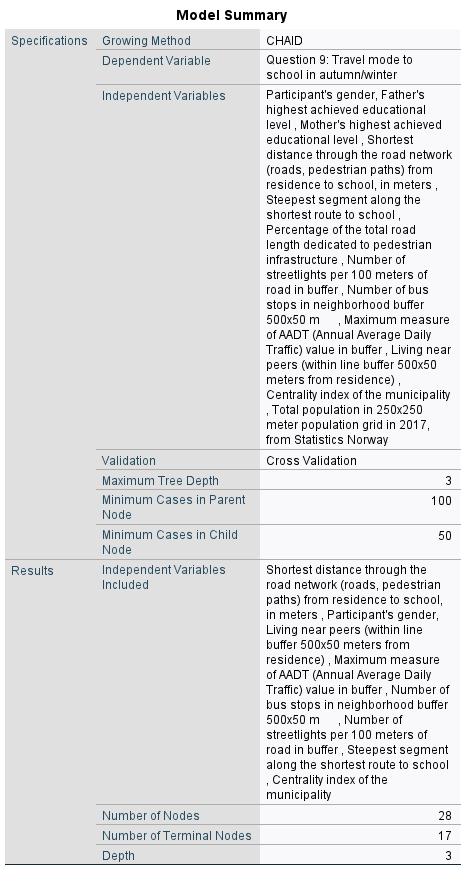

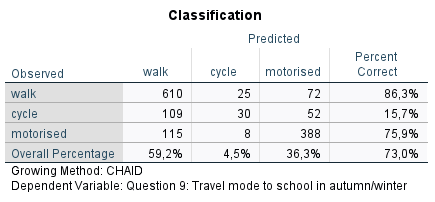

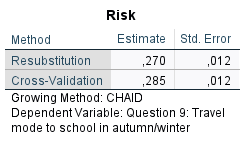


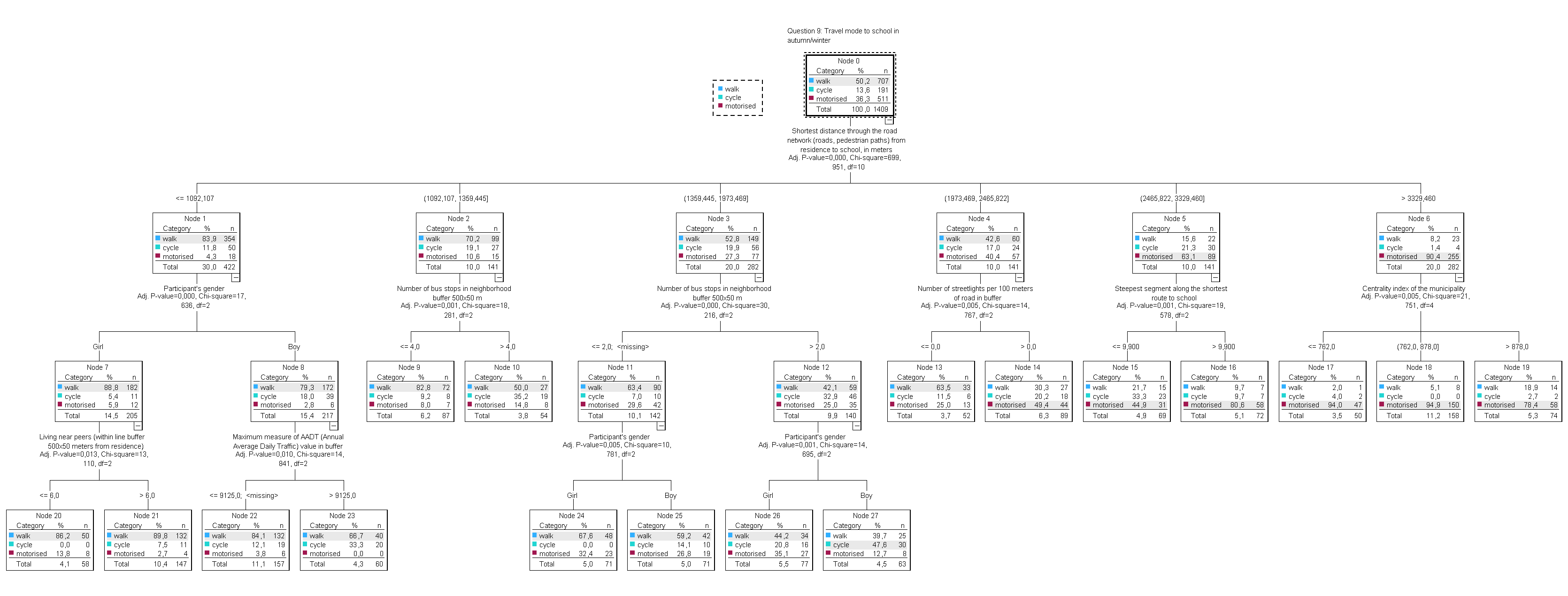


**4.0 Syntax for Decision Tree analysis using CRT and QUEST algorithm**

**Note**: The default setting in SPSS is 3 for CHAID and 5 for CRT and QUEST. A comparison between CHAID with a maximum of 3 layers and QUEST and CRT with a maximum of 5 layers showed predictive accuracies of 71%, 69%, and 49%, respectively. In the syntax and results below, the maximum depth is set to 3 layers for both QUEST (55%) and CRT (67%) in comparison with CHAID at 3 layers (71%), described above.

| **Syntax CRT algorithm** (summer model) | **Syntax QUEST algorithm** (summer model) |
| --- | --- |
| TREE Q7_outcome [n] BY Gender [n] Education_father [n] Education_mother [n] Distance_Road [s]  Steepest_hill [s] Pedestrian_share [s] Streetlights [s] Count_busstop_buffer [s]  Trafic_exposure_max [s] Peers_nearby [s] Rural_Urban_index [s] Population [s]  /TREE DISPLAY=TOPDOWN NODES=STATISTICS BRANCHSTATISTICS=YES NODEDEFS=YES SCALE=AUTO  /DEPCATEGORIES USEVALUES=[1.00 2.00 3.00]  /PRINT MODELSUMMARY CLASSIFICATION RISK  /METHOD TYPE=CRT MAXSURROGATES=AUTO PRUNE=NONE  /GROWTHLIMIT MAXDEPTH=3 MINPARENTSIZE=100 MINCHILDSIZE=50  /VALIDATION TYPE=CROSSVALIDATION(10) OUTPUT=BOTHSAMPLES  /CRT IMPURITY=GINI MINIMPROVEMENT=0.0001  /COSTS EQUAL  /PRIORS FROMDATA ADJUST=NO  /MISSING NOMINALMISSING=MISSING. | TREE Q7_outcome [n] BY Gender [n] Education_father [n] Education_mother [n] Distance_Road [s]  Steepest_hill [s] Pedestrian_share [s] Streetlights [s] Count_busstop_buffer [s]  Trafic_exposure_max [s] Peers_nearby [s] Rural_Urban_index [s] Population [s]  /TREE DISPLAY=TOPDOWN NODES=STATISTICS BRANCHSTATISTICS=YES NODEDEFS=YES SCALE=AUTO  /DEPCATEGORIES USEVALUES=[1.00 2.00 3.00]  /PRINT MODELSUMMARY CLASSIFICATION RISK  /METHOD TYPE=QUEST MAXSURROGATES=AUTO PRUNE=NONE  /GROWTHLIMIT MAXDEPTH=3 MINPARENTSIZE=100 MINCHILDSIZE=50  /VALIDATION TYPE=CROSSVALIDATION(10) OUTPUT=BOTHSAMPLES  /QUEST ALPHASPLIT=0.05  /COSTS EQUAL  /PRIORS FROMDATA ADJUST=NO  /MISSING NOMINALMISSING=MISSING. |

**4.1 Model performance for CRT algorithm - summer season (Q7_outcome)**


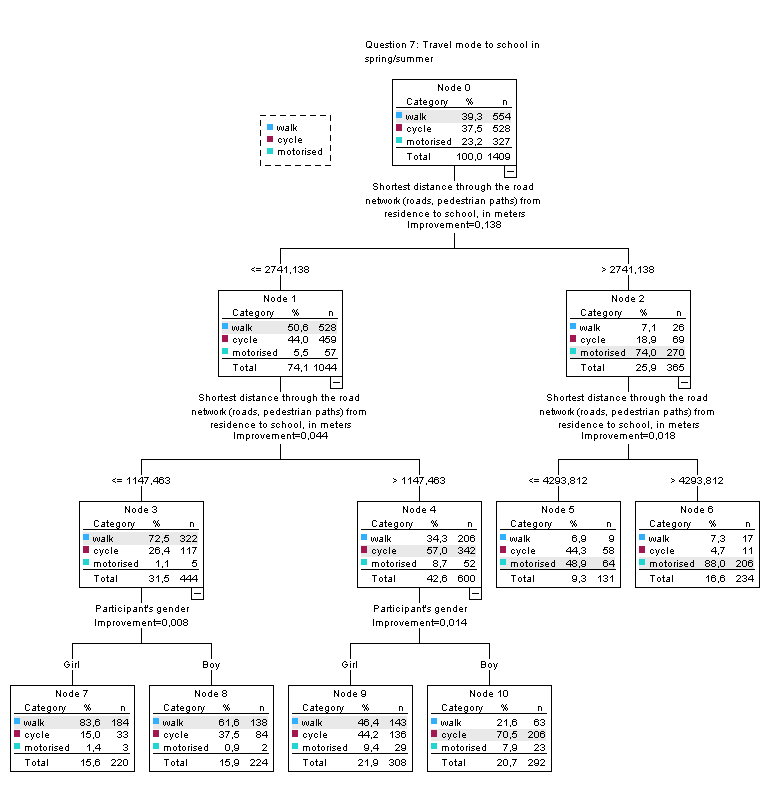


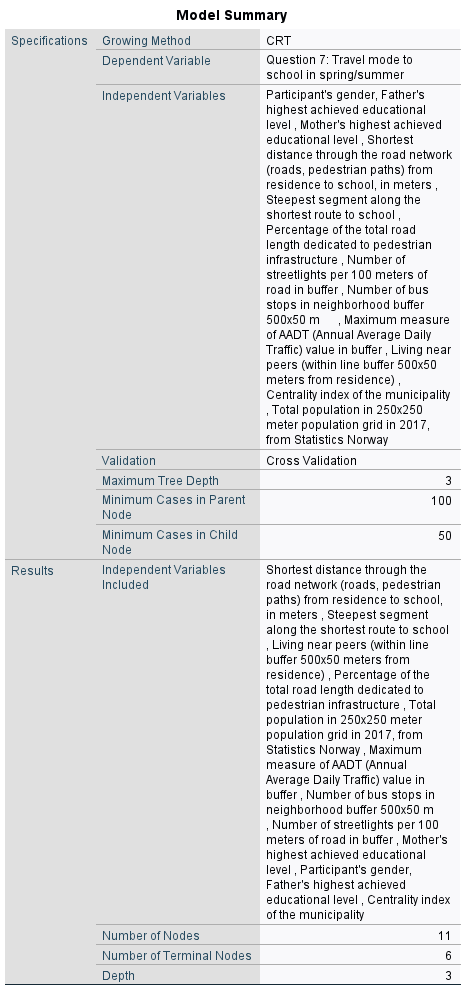

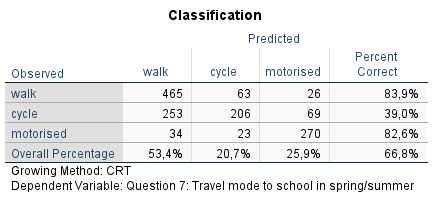

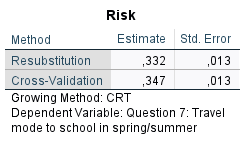


**4.2 Model performance for QUEST algorithm - summer season (Q7_outcome)**

**
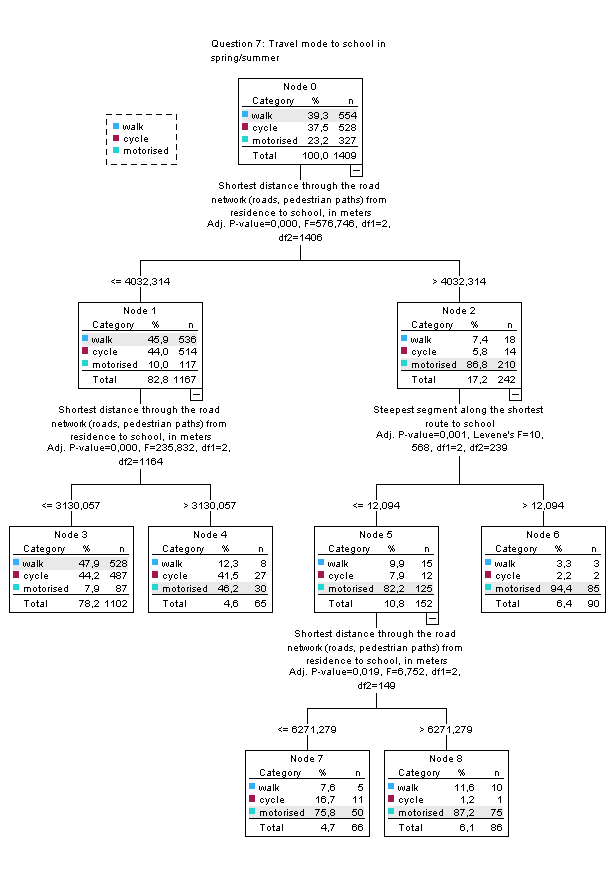
**

**
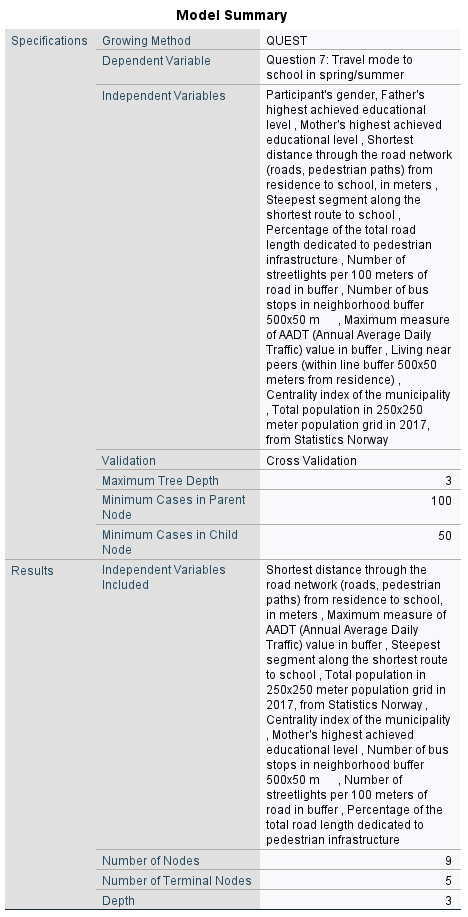

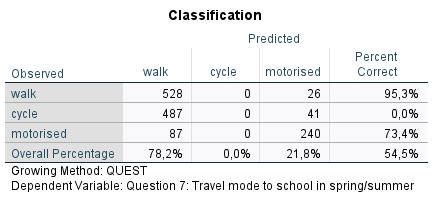

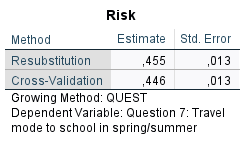
**
