# Supplementary material for: Objectively measured environmental factors in relation to school travel mode among adolescents: a decision tree analysis
Source: Int J Behav Nutr Phys Act. 2025 Mar 4;22:26. doi: 10.1186/s12966-025-01727-6 (PMC11877687; doi:10.1186/s12966-025-01727-6)
Supplement: Supplementary file 2 — Supplementary Material 2 [file 12966_2025_1727_MOESM2_ESM.docx]

**Appendix B: Results of a chi-square sensitivity analysis**

This file contains the results of a chi-square sensitivity analysis comparing the included sample with the total sample from the main project. The data analyzed using Statistical Package for Social Sciences software (IBM SPSS Statistics for Windows, Version 22.0. Armonk, NY: IBM Corp. USA), and the significance level was set to p < 0.05.

No significant differences were detected between the groups in spring/summer or autumn/winter season. The analysis was conducted to ensure that the findings from the included sample are representative of the overall population. For reproducibility purposes, the SPSS syntax used for the chi-square analysis is also provided.

**Results from chi square test on travel mode to school in the spring/summer season:**

|  | **Walk** | **Cycle** | **Bus/Tram/Boat** | **Car/Motorcycle/Moped** | **Total** |
| --- | --- | --- | --- | --- | --- |
| **Included sample** | 554 (39,3%) | 528 (37,5%) | 270 (19,2%) | 57 (4,0%) | 1409 (100%) |
| **ScIM project** | 666 (38,3%) | 634 (36,5%) | 364 (20,9%) | 75 (4,3%) | 1739 (100%) |
| **Total** | 1220 (38,8%) | 1162 (36,9%) | 634 (20,1%) | 132 (4,2%) | 3148 (100%) |

|  | **Value** | **df** | **Asymptotic Significance (2-sided)** |
| --- | --- | --- | --- |
| Pearson Chi-Square | 1,769^a^ | 3 | ,622 |
| Likelihood Ratio | 1,773 | 3 | ,621 |
| Linear-by-Linear Association | 1,153 | 1 | ,283 |
| N of Valid Cases | 3148 |  |  |

a. 0 cells (0,0%) have expected count less than 5. The minimum expected count is 59,08. df = degrees of freedom.

**Results from chi square test on travel mode to school in the autumn/winter season:**

|  | **Walk** | **Cycle** | **Bus/Tram/Boat** | **Car/Motorcycle/Moped** | **Total** |
| --- | --- | --- | --- | --- | --- |
| **Included sample** | 707 (50,2%) | 191 (13,6%) | 343 (24,3%) | 168 (11,9%) | 1409 (100%) |
| **ScIM project** | 852 (48,9%) | 224 (12,9%) | 454 (26,1%) | 212 (12,2%) | 1742 (100%) |
| **Total** | 1559 (49,5%) | 415 (13,2%) | 797 (25,3%) | 380 (12,1%) | 3151 (100%) |

|  | **Value** | **df** | **Asymptotic Significance (2-sided)** |
| --- | --- | --- | --- |
| Pearson Chi-Square | 1,489^a^ | 3 | ,685 |
| Likelihood Ratio | 1,491 | 3 | ,684 |
| Linear-by-Linear Association | ,767 | 1 | ,381 |
| N of Valid Cases | 3151 |  |  |

a. 0 cells (0,0%) have expected count less than 5. The minimum expected count is 169,92. df = degrees of freedom.

**Syntax for both analyses:**

CROSSTABS

/TABLES=Sample_2 BY s_7_t1_m

/FORMAT=AVALUE TABLES

/STATISTICS=CHISQ

/CELLS=COUNT ROW

/COUNT ROUND CELL.

CROSSTABS

/TABLES=Sample_2 BY s_9_t1_m

/FORMAT=AVALUE TABLES

/STATISTICS=CHISQ

/CELLS=COUNT ROW

/COUNT ROUND CELL.
