# Supplementary material for: Objectively measured environmental factors in relation to school travel mode among adolescents: a decision tree analysis
Source: Int J Behav Nutr Phys Act. 2025 Mar 4;22:26. doi: 10.1186/s12966-025-01727-6 (PMC11877687; doi:10.1186/s12966-025-01727-6)
Supplement: Supplementary file 3 — Supplementary Material 3 [file 12966_2025_1727_MOESM3_ESM.docx]

**Appendix C: Correlation coefficients between independent variables (ordinal and scale measurement level)**

**Table:** Associations between predictor variables. Data presented as spearman’s r. Significant associations are highlighted in bold.

|  | **Built environmental** | | | | | | | | **Parents education** | |
| --- | --- | --- | --- | --- | --- | --- | --- | --- | --- | --- |
|  | Steep hill | Population | Bus stop | Centrality index | Streetlights | Peers nearby | Pedestrian infrastructure | Traffic Exposure | Father | Mother |
| Distance | **0,40** | **-0,42** | **-0,32** | **-0,19** | **-0,21** | **-0,50** | **-0,38** | **-0,30** | **-0,06** | 0,00 |
| Steep hill |  | -0,04 | **0,08** | **0,13** | **0,12** | **-0,06** | **-0,10** | 0,03 | 0,03 | **0,10** |
| Population |  |  | **0,38** | **0,41** | **0,30** | **0,64** | **0,50** | **0,42** | 0,08 | 0,00 |
| Bus stop |  |  |  | **0,09** | **0,42** | **0,40** | **0,36** | **0,38** | **0,06** | 0,03 |
| Centrality Index |  |  |  |  | **0,09** | **0,29** | **0,35** | **0,46** | 0,02 | -0,01 |
| Streetlights |  |  |  |  |  | **0,27** | **0,19** | **0,46** | 0,01 | -0,01 |
| Peers nearby |  |  |  |  |  |  | **0,44** | **0,30** | **0,15** | 0,05 |
| Pedestrian infrastructure |  |  |  |  |  |  |  | **0,38** | 0,05 | -0,03 |
| Traffic exposure |  |  |  |  |  |  |  |  | **0,06** | 0,00 |
| Education Father |  |  |  |  |  |  |  |  |  | **0,42** |

Cases with missing values were excluded listwise, N = 1156. Similar associations were made with pairwise exclusion of missing data.
